# A comparison of SNP and STR loci for delineating population structure and performing individual genetic assignment

**DOI:** 10.1186/1471-2156-11-2

**Published:** 2010-01-06

**Authors:** Kevin A Glover, Michael M Hansen, Sigbjørn Lien, Thomas D Als, Bjørn Høyheim, Øystein Skaala

**Affiliations:** 1 Institute of Marine Research, PO Box 1870, Nordnes N- 5817 Bergen, Norway; 2 Department of Biological Sciences, Aarhus University, Ny Munkegade 114, DK-8000 Aarhus C, Denmark; 3 Norwegian University of Life Science, Centre of Integrative Genetics and Department of Animal and Aquacultural Sciences, PO Box 5003, 1430 Ås, Norway; 4 Technical University of Denmark, National Institute of Aquatic Resources, Section for Population Genetics, Vejlsøvej 39, DK-8600, Silkeborg, Denmark; 5 Norwegian School of Veterinary Science, BasAM-Genetics, PO Box 8146, 0033 Oslo, Norway

## Abstract

**Background:**

Technological advances have lead to the rapid increase in availability of single nucleotide polymorphisms (SNPs) in a range of organisms, and there is a general optimism that SNPs will become the marker of choice for a range of evolutionary applications. Here, comparisons between 300 polymorphic SNPs and 14 short tandem repeats (STRs) were conducted on a data set consisting of approximately 500 Atlantic salmon arranged in 10 samples/populations.

**Results:**

Global F_ST _ranged from 0.033-0.115 and -0.002-0.316 for the 14 STR and 300 SNP loci respectively. Global F_ST _was similar among 28 linkage groups when averaging data from mapped SNPs. With the exception of selecting a panel of SNPs taking the locus displaying the highest global F_ST _for each of the 28 linkage groups, which inflated estimation of genetic differentiation among the samples, inferred genetic relationships were highly similar between SNP and STR data sets and variants thereof. The best 15 SNPs (30 alleles) gave a similar level of self-assignment to the best 4 STR loci (83 alleles), however, addition of further STR loci did not lead to a notable increase assignment whereas addition of up to 100 SNP loci increased assignment.

**Conclusion:**

Whilst the optimal combinations of SNPs identified in this study are linked to the samples from which they were selected, this study demonstrates that identification of highly informative SNP loci from larger panels will provide researchers with a powerful approach to delineate genetic relationships at the individual and population levels.

## Background

The characterisation and availability of single nucleotide polymorphisms (SNPs) in non-model organisms is increasing rapidly [1,2], and within the field of population genetics, growing attention is being given to this class of marker to address a broad range of evolutionary questions (reviewed by [3,4]).

Highly polymorphic short tandem repeat loci (STR), commonly known as microsatellites, have been the primary molecular tool of choice for addressing evolutionary questions for nearly two decades. However, these markers display several negative characteristics including size homoplasy [5], complex mutational patterns, and are prone to genotyping errors [6]. Furthermore, STR scoring is platform dependant [7], making inter-laboratory collaboration a challenge.

In contrast to STR analysis, SNP genotyping reveals polymorphisms directly in the DNA sequence, circumventing the need for between laboratory calibration. Furthermore, development of high through-put genotyping platforms permits simultaneous genotyping of thousands of loci, enabling the identification of highly diagnostic panels [8]. SNPs occur throughout the genome, and thus offer the possibility for detailed information for all regions, which is an advantage in identifying genes under selection or when mapping genes related to specific traits. Nevertheless, the implementation of SNPs to delineate population genetic structure is still in its infancy outside the field of human genetics (but see SNPs in cattle e.g., [9-11]) for example where they have been demonstrated to out-perform microsatellites for specific questions such as individual ancestry [8].

Several non-human population genetic studies have compared results obtained from STR and SNP data sets, however, many comparisons have involved relatively low numbers of SNP loci [12-16]. Consequently, few non-human genetic studies have been able to effectively investigate how selecting a "highly informative" set of diagnostic loci from a larger pool, for example by selecting those displaying highest global F_ST_, may influence and potentially bias genetic relationships among the populations being studied. Furthermore, few studies have compared the power of SNPs and STRs to perform genetic assignment of individuals to populations (but see [15,16]), but once again, only with modest numbers of loci. The latter point is important because whereas genetic relationships may be effectively delineated with even low to moderate numbers of SNP loci (e.g., [17,18], the accuracy of genetic assignment may be linked to the number of independent alleles [19-22], although this is not necessarily true when comparing between marker classes [16], or where large resources of SNPs have been scanned for "highly diagnostic" loci to perform assignment [8].

Individual genetic assignment is an important tool in the management of domesticated and wild genetic resources, and has been used in forensic cases to detect illegal translocations of animals [23,24], illegal trade [25], fraud [26] and source of origin for escaped domesticated animals [27]. Furthermore, assignment tests have been used in the investigation of evolutionary processes in addition to the identification of hybrids [28] and species[29].

The aim of the present study was to compare the performance of a large resource of SNPs (388) and a panel of STRs (15) to perform individual genetic assignment and delineate population structure. This was achieved through genotyping a set of Atlantic salmon (*Salmo salar *L.) samples originating from a number of fish farms in Norway, and, a number of escaped farmed fish. This species was chosen due to the fact that a large number of verified SNPs have previously been identified and mapped [1,2], and, that a high through-put genotyping platform for the analysis of the SNPs existed.

## Methods

### Biological samples

Domesticated Atlantic salmon can escape from aquaculture facilities into the wild, and in the period 2001-2005, 260 000-715 000 farmed escapees were officially reported in Norway to the Directorate of Fisheries annually. However, the true number of escapees is probably higher due to underreporting [30]. In an attempt to assist the Norwegian authorities in improving regulation over the aquaculture industry, the Institute of Marine Research in Norway developed a DNA based forensic method to identify escaped salmon to farm of origin [22,27]. In short, this method is based upon screening a panel of STR loci on samples of escaped salmon that are recaptured in the wild in addition to salmon collected from farms in the surrounding area that are considered as potential sources of the escapees. A combination of genetic assignment in addition to probability based exclusion is used to identify the most likely source(s) of origin for the escapees.

The data set chosen for analysis in the present study consisted of approximately 500 Atlantic salmon resulting from investigating an unreported escapement episode. Fish were sampled from nine cages located on six marine farms (hereon referred to as samples A-I), in addition to 50 farmed escapees (RF) that had been recaptured in the vicinity of these farms. The recaptured fish were distinguished as farmed salmon based upon morphological characteristics. The farms, locations and exact dates of collection remain anonymous for legal reasons.

### STR analysis

DNA extraction and STR analysis was performed at the Institute of Marine Research (IMR) in Bergen. DNA extraction was carried out in 96 well format using a Qiagen DNAeasy kit according to the manufacturers' instructions. Each plate contained a minimum of two negative controls. DNA was extracted twice for 48 of the 50 escapees (separate dates). The following fifteen STR loci were amplified in three separate multiplex PCR reactions; SSsp3016 (Genbank no. AY372820), SSsp2210, SSspG7, SSsp2201, SSsp1605, SSsp2216 [31], Ssa197, Ssa171, Ssa202 [32], SsaD157, SsaD486, SsaD144 [33], Ssa289, Ssa14 [34], SsaF43 [35], using a modification of a previously described protocol [36]. These loci are routinely used for performing Atlantic salmon genetics studies at IMR but do not represent an optimised set of loci for performing genetic assignment of farmed escapees. Locus SsaD486 was monomorphic in the entire data set and was excluded from all statistical analyses. PCR products were analysed on an ABI 3730 Genetic Analyser using the 500 LIZ™ size-standard. Alleles were automatically binned and manually checked in the Genotyper software prior to data analysis. A total of 87 individuals (from individual samples displaying partial PCR amplification failure on first amplification/electrophoresis) in addition to 48 of the 50 escapees were re-analysed (pcr amplification then electrophoresis). These individuals served as a genotyping controls. Several authors [6,37] have recommended the routine use of genotyping controls in genetic data sets to estimate error rates.

### SNP screening

SNP genotyping was performed using the MassARRAY platform from Sequenom (San Diego, USA). A total of 388 SNPs were included in the study. Map position and flanking sequence of the majority of these SNPs are from Moen et al. [2] and Lorenz et al. [38] (Additional file 1). Multiplexes and primer sequences for genotyping are available upon request. All SNP genotyping was performed according to the iPLEX protocol (available at http://www.sequenom.com) using the MassARRAY™ Analyzer (Autoflex mass spectrometer) from Sequenom. Genotypes were assigned in real time [39] by using the MassARRAY SpectroTYPER RT v3.4 software followed by manual inspection of genotypes using the MassARRAY TyperAnalyzer v3.3 software.

### Statistical analysis

In order to compare the two classes of markers, the STR and SNP data sets were mixed in various combinations. These are described in the results section as some combinations were test specific. However, the following four data sets were used as the start point for the majority of the analyses within: 1) 14 STR loci, 2) 300 SNP loci, 3) 28 SNP loci (selecting the SNP displaying the highest global F_ST _for each of the 28 linkage groups), and 4) 195 mapped SNPs with minimal distance of > 1 cM to nearest SNP (selecting the SNP displaying the highest global F_ST _for 2 or more SNPs < 1 cM). For some tests, loci were ranked prior to computation. Ranking of loci was carried out by three methods including number of alleles displayed through samples A-I (STR loci only), global F_ST _across samples A-I (STR and SNP loci), and with the locus assignment power program BELS across samples A-I [40]. BELS was programmed to maximise mean individual assignment power on the data set without any re-sampling.

Allelic variation, heterozygosity and F statistics were computed in the program MSA [41]. Arlequin V3.11 [42] was used to calculate deviation from Hardy Weinberg Equilibrium. MEGA [43] was used to produce phylogenetic trees for the various data sets using the UPGMA method on matrices of pair-wise F_ST _values. The trees were linearised assuming equal evolutionary rates in all lineages [44]. Geneclass V. 1.02 [45] was used to perform self-assignment simulations among samples A-I using the leave one out sub-option, and direct assignment of the escapees to these samples. All tests were performed using the Rannala and Mountain [46] method of estimating allele frequencies unless otherwise stated.

Bayesian clustering analysis implemented in STRUCTURE 2.2 [47,48] was used for estimating the number of populations/groups (*k*) represented by the data set. Following pilot analysis, main runs assuming *k *= 1-6, each with 3-5 iterations (depending upon data set), were conducted in order to estimate the most likely *k *and to assign individuals to these groups without using prior information about their sample of origin using correlated allele frequencies and an admixture model. Each run consisted of a burn-in of 100,000 MCMC steps, followed by 500,000 replications. STRUCTURE 2.2 was also used to perform a modified self-assignment procedure by removing 10 individual salmon at random from samples A, D, F and G, then assigning these individuals to the baseline which did not include those individuals. This was conducted by using the prior population information for the baseline samples and no prior population information for these 40 individuals. The results obtained from this analysis were compared to an identical procedure in Geneclass for these 40 individuals (in the latter case removing the 40 fish from the baseline file and entering them into a separate file of "unknown" individuals).

## Results

### Genotyping quality

DNA was isolated from a total of 512 salmon that were analysed for 15 STR loci at IMR and 388 SNP loci at CIGENE. In the two laboratories, 13 and 14 of the individual DNA extracts failed to yield PCR products for any of the loci, leaving STR and SNP data sets consisting of 499 and 498 individual fish respectively. Individuals failing to yield PCR products for the STR loci were spread among samples, whereas all complete amplification failures in the SNP data set were observed within the sample of escapees.

Within the STR data set, a total of 87 individuals displaying PCR failure in ≥ 2 loci were selected for re-amplification for all STR loci in order to increase the scoring percentage in the data set. These individuals represented the majority of, but not all the individuals displaying PCR failure at ≥ 2 loci. As a result of re-analysing these 87 individuals, in addition to analysis of the second DNA isolate for 48 of the 50 escapees, > 1000 genotypes in 135 fish were independently scored on two occasions. Of these, no genotyping inconsistencies were observed between original and re-analysis. The resultant overall genotyping success in the STR data set (n = 499 × 15 markers) was > 99% (Additional file 1).

Within the SNP data set consisting of 388 loci × 498 individuals, amplification of individual loci was highly variable, ranging from 0-100% scoring (Additional file 1). Loci displaying less than 95% amplification in the data set were excluded (n = 79). Of the remaining 309 loci analysed for 498 fish, overall genotyping success was > 98%, whereas genotyping success ranged from 66%-100% for loci across individual fish. Nine of the 309 loci were monomorphic in all samples. These loci were excluded from all further analyses, leaving a complete SNP data set of 300 polymorphic loci.

### Within-sample variation

Within the SNP data set, 104 out of 2706 tests of HWE were significant at *α *= 0.05 (= 3.8%). A total of 294 tests were not computed due to some loci being monomorphic in some samples. Following application of Bonferroni correction in a conservative manner (*α *= 0.05/300 loci = 0.00017), only 6 of the observed deviations remained significant. These were observed in (locus:sample) 74:A, 202:E, 202:F, 202:G, 207:D, 300:C. Within the STR data set, 16 out of 140 tests of HWE were significant at *α *= 0.05 (11.4%). These deviations were spread among loci and samples, with all being implicated in a minimum of one significant deviation except loci *Ssa289*, *SsaF43, SSspG7*, and samples E, F. Following application of Bonferroni correction in a conservative manner (*α *= 0.05/14 = 0.0036), none of these deviations remained significant.

A summary of the allelic variation and expected average heterozygosity per sample are presented (Table 1). In total, 600 alleles were observed at the 300 SNP loci whilst 179 alleles were observed at the 14 STR loci. The percentage of the total number of alleles observed within specific samples varied between 92-98% and 53-74% for the SNP and STR data sets respectively. Despite large differences in absolute numbers of alleles between marker sets, corroboration between allelic variation for individual samples (relative to other samples) was observed between marker classes. Expected heterozygosity averaged over loci and samples varied greatly between the 300 SNP data set (He = 0.32) and the 14 STR loci (He = 0.78), although expected heterozygosity, relative to the other samples was similar between marker classes.

**Table 1:** Summary of within sample genetic variation measured by absolute number of alleles and expected heterozygosity (He) for 300 SNP and 14 STR loci.

Sample	300 SNP loci			14 STR loci		
	A_T_	A_M_	He	A_T_	A_M_	He
A	579	1.93	0.29	116	8.3	0.72
B	565	1.89	0.29	104	7.4	0.72
C	552	1.84	0.28	94	6.7	0.69
D	571	1.90	0.29	102	7.3	0.73
E	551	1.84	0.29	99	7.1	0.71
F	563	1.88	0.29	99	7.1	0.71
G	576	1.92	0.31	111	7.9	0.71
H	578	1.93	0.31	126	9.0	0.77
I	576	1.92	0.32	120	8.6	0.74
RF	585	1.95	0.31	118	8.4	0.76

Total	600	2.0	0.32	179	12.8	0.78

A_T _= total number of alleles observed, A_M _= mean number of alleles observed per locus.

### Among sample genetic differentiation

**Figure 1:**
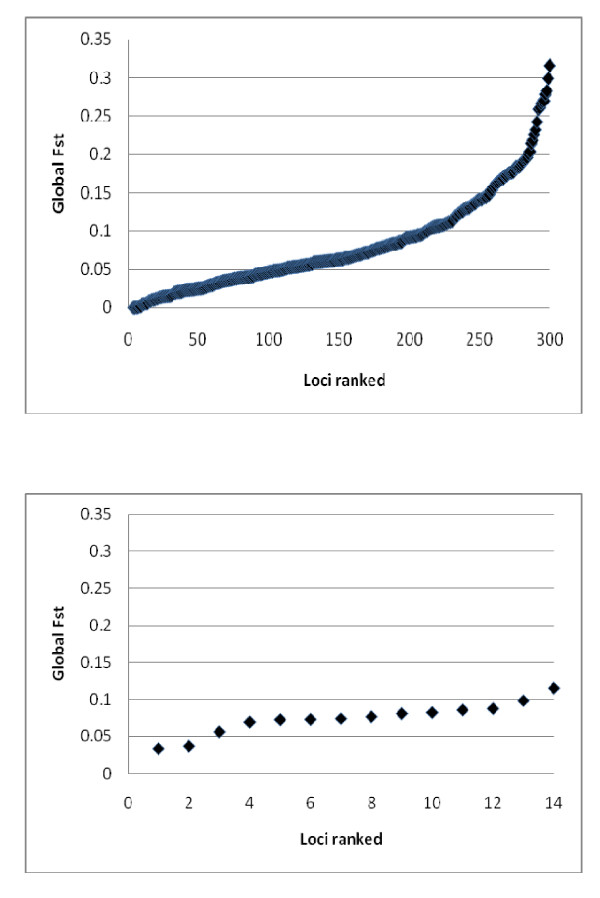
**Global F_ST _observed among 9 samples/populations of farmed salmon based upon 300 SNP loci (top), and 14 STR loci (bottom)**.

Global F_ST _values per locus ranged from 0.033-0.115 among the 14 STR loci, and -0.002-0.316 among the 300 SNP loci (Figure 1). 87 of the 300 SNP loci (= 29%) displayed global F_ST _values over 0.1 whereas two STR loci (*SsaD157 *and *SSsp2210*) exceeded a global F_ST _of 0.1 (= 14%). Despite the considerable differences in F_ST _among the SNP loci, an analysis using the Bayesian simulation-based test by Foll and Gaggiotti [49] only identified a single SNP as an outlier (Bayesian *p *< 0.01; data not shown). Hence, there was limited evidence to suggest that the loci might be under diversifying selection in the analysed set of samples.

Within each linkage group, global F_ST _per SNP locus varied greatly (Additional file 2). For example, global F_ST _ranged between 0.013-0.316 per locus on linkage group d03. Eleven of the linkage groups consisted of 10 or more SNP loci. When mean global F_ST _per linkage group was compared among them, no significant differences were observed (Kruskal-Wallis non-parametric ANOVA: *P *= 0.12). Analysis with a parametric ANOVA gave a similar result (P = 0.4). The majority of tightly linked loci (i.e., those located within the same contig and < 0.1 cM distance) displayed very similar global F_ST _values to each other, however, this was not universally true. For example, whilst the three loci located at 13.7 cM on linkage group d02 displayed global F_ST _values of 0.026-0.034, and the two loci located at 3 cM on linkage group d14 displayed global F_ST _values of 0.0002 and 0.0003, the two loci located at 61.8 cM on linkage group d06 displayed highly contrasting global F_ST _values of 0.05 and 0.27.

**Figure 2:**
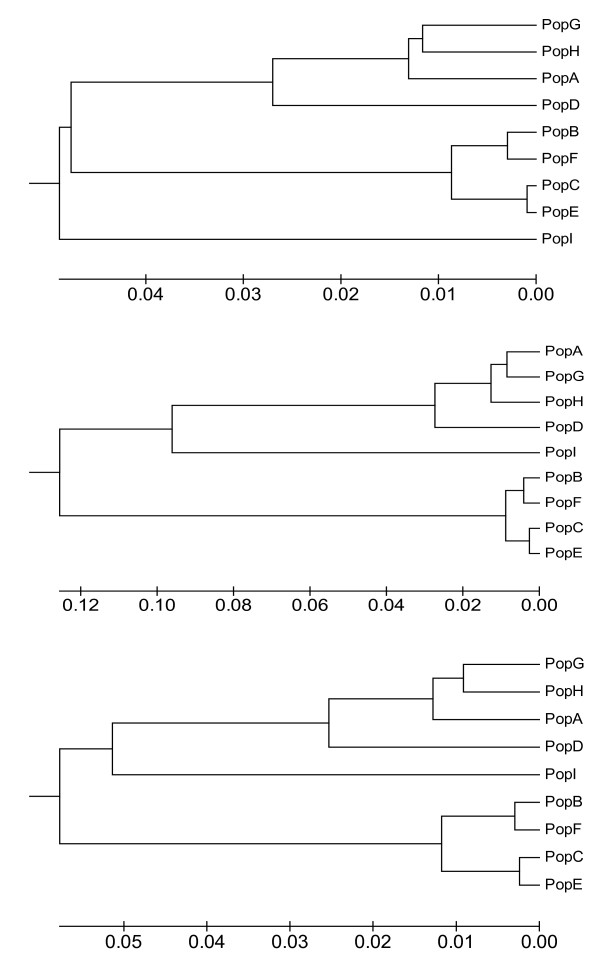
**Genetic relationship among 9 samples/populations of farmed Atlantic salmon calculated with 14 STR loci (top), 28 unlinked SNPs taking the SNP displaying highest global F_ST _per linkage group (middle), and 300 SNPs (bottom)**. The optimal tree is presented using an UPGMA method with optimal sum of branch length calculated as 0.12 (top), 0.41 (middle) and 0.23 (bottom).

The genetic relationships between the nine samples collected from farms are presented as UPGMA diagrams (Figure 2). The data set consisting of one SNP per linkage group, selected by highest global F_ST_, displayed greater among-sample differentiation than other data sets, however, among sample relationships were remarkably similar for all data sets (Figure 2), including those consisting of all 314 markers combined and 195 mapped SNPs (data not presented).

### Self-assignment simulations

**Figure 3:**
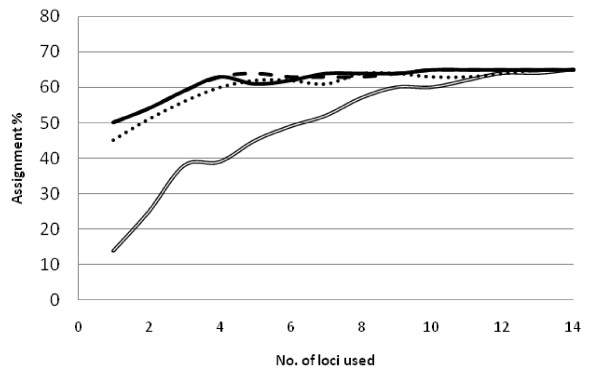
**Correct self-assignment percentage plotted against cumulative number of STR loci selecting loci displaying the highest global F_ST _(dotted line), highest number of alleles (solid line), least number of alleles (double line), and greatest assignment power when ranked by the program BELS (broken line)**. Overall assignment reached a maximum of 65%.

**Figure 4:**
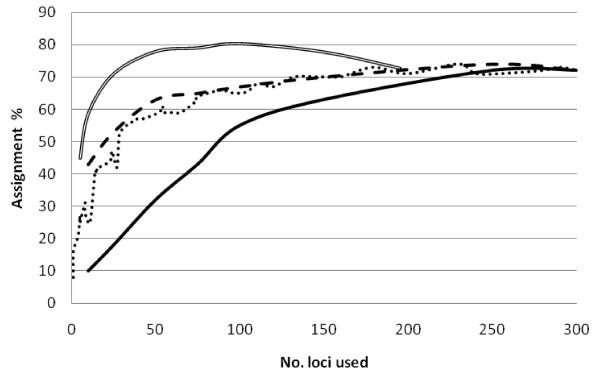
**Correct self-assignment percentage plotted against cumulative number of SNP loci selecting loci randomly (dotted line), the most informative 10, 25, 50, 75, 100, 150, 250 and all 300 loci sorted by global F_ST _(broken line), the least informative 10, 25, 50, 75, 100, 150, 250 and all 300 loci sorted by global F_ST _(solid line), and the most informative 5, 10, 25, 50, 75, 100, 150, 195 loci sorted by the program BELS taking only mapped loci > 1 cM distance from each other (double line)**.

Using Geneclass, the overall accuracy of self-assignment was 65%, 73% and 73% for the data sets consisting of 14 STR, 300 SNPs and 195 mapped SNPs respectively (Figures 3 and 4). In the STR data set, with the exception of selecting loci starting with the least polymorphic first, the various selection methods only gave small differences in increase of assignment with number of loci (Figure 3), and, almost no further gain in assignment was observed past four loci. In the 300 SNP data set, large differences in the cumulative assignment curve were observed between the different selection methods (Figure 4), furthermore, selection of loci from the 195 mapped SNPs gave the highest overall assignment when approximately 100 loci were included in the analysis (80% assignment). Past this number of loci, the assignment accuracy dropped. Comparing the two marker types, the "best" 15 SNPs selected by BELS matched the level of assignment achieved by the best 4 STR loci selected by allelic variation (and BELS).

When self-assignment simulations were conducted with the SNP loci displaying the highest global F_ST _per linkage group (n = 28), overall assignment reached 58% which is similar to the value reported for the best 25 SNP loci selected by global F_ST _irrespective of linkage group. However, as the SNP loci displaying highest global F_ST _values were spread between linkage groups (Additional file 1), these two sets displayed considerable locus overlap.

**Figure 5:**
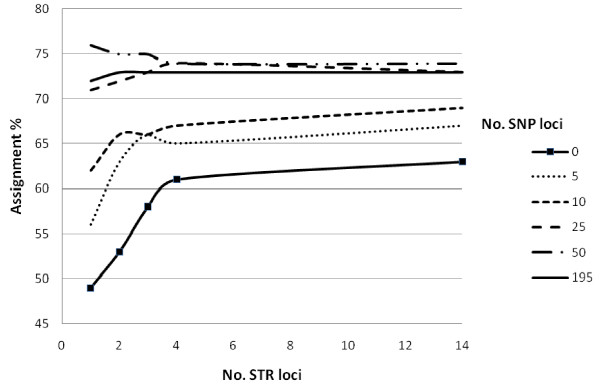
**Correct self-assignment percentage plotted against number of STR loci, taking loci displaying greatest number of alleles first, when combining each STR set with 0, 5, 10, 25, 50 and 195 SNPs starting with the most informative SNPs ranked by the program BELS**. Integrated figure legend shows number SNP loci added.

**Figure 6:**
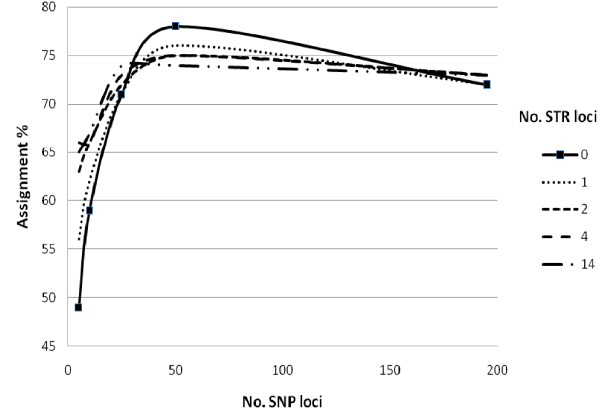
**Correct self-assignment percentage plotted against number of SNP loci taking the most informative loci ranked by the program BELS first, when combining each SNP set with 0, 1, 2, 4 and all 14 STR loci taking the STR loci displaying greatest number of alleles first**. Integrated figure legend shows number of STR loci added.

Addition of 1-4 STR loci increased assignment for data sets starting with 5, 10 and 25 SNPs selected by BELS, however, for the data set starting with 50 SNPs, addition of STR loci lead to a reduction in assignment (Figure 5). When selecting SNP loci based upon global F_ST_, addition of 1-4 STR loci increased assignment in data set starting with up to 100 loci, although a drop in overall assignment was observed when starting with 300 SNP loci (data not presented). For all data sets starting with different numbers of STR loci, addition of up to 50 SNP loci increased assignment when selecting loci with BELS (Figure 6), and global F_ST _(data not presented).

"Self-assignment" of the 40 individuals removed from the baseline (10 from A, D, F, G) revealed identical results between the programs Geneclass and STRUCTURE for data sets consisting of 28 SNPs (58%), and 195 SNPs (78%). The latter is an important as STRUCTURE used a marker linkage model, taking marker distance into the computations, whereas Geneclass treated the loci as independent. STRUCTURE outperformed Geneclass for self-assignment of these 40 individuals using 14 STR loci (73% contra 65%), and all 300 SNPs (88% contra 80%).

The absolute accuracy of assignment was lower when computed using a distance based calculation [50], however, the trends in assignment when mixing marker classes were very similar to the trends reported above, although no drop in assignment was observed when STR loci were added to the data set starting with 50 SNP loci.

### Assignment of the escapees

Direct assignment (Table 2) using all variants of the STR and SNP data sets (including all combinations) demonstrated that nearly all of the escapees originated from sample I. Whilst only a low number of loci were required to directly assign most of the escapees to the sample I, simulations of exclusion from each sample rejecting at *P *= 0.01 indicated that more loci were required for exclusion of the alternative samples, and, this trend was evident for both marker classes and marker selection criteria (data not presented).

**Table 2:** Direct assignment of the escapees to sample for different sets of loci.

SNPs					
Sample	10 SNP	25 SNP	28 unlinked SNPs	195 mapped SNPs	300 SNPs
	
I	32	34	34	35	35
A-H	4	2	2	1	1
STRs					
Sample	2 STR		4 STR	14 STR	
	
I	42		42	47	
A-H	6		6	1	

STR and SNP combined					
Sample	4STR + 10 SNP		4 STR + 25 SNP	14 STR + 300 SNP	
	
I	46		47	49	
A-H	3		2	0	

SNP loci were selected by highest global F_ST _whereas STRs were selected by number of alleles. 28 unlinked SNPs = highest global F_ST _for each of the linkage groups. 195 mapped SNPs only includes SNPs > 1 cM distance from next SNP. Different total numbers of escapees in the different data sets is due to differences in numbers of individuals genotyped.

### Bayesian clustering of the data set

For the data sets consisting of 14 STR, 28 SNP, 195 SNP and 300 SNP loci, *k *was estimated at 4 or 5 (data not presented), and consequently, assignment of the individuals is presented for *k *= 3-5 (Additional file 3). The inter-sample relationships revealed by STRUCTURE 2.2 displayed concordance with the UPGMA diagrams for these data sets (Figure 2), furthermore, with minor differences, all four data sets examined displayed a similar pattern of relationships between samples, for each *k*. These analyses clearly linked the escapees (RF) and sample I into a single cluster separate from all other samples, confirming the assignment results conducted above.

## Discussion

Comparisons between marker classes to perform genetic assignment can be conducted in a number of ways, although locus by locus, total number of alleles, or cost per information unit comparisons are commonly applied. In this study, it took 15 of the best SNP loci (total 30 alleles) to match the accuracy of self-assignment achieved by the four most polymorphic STR loci (83 alleles), clearly demonstrating that although some of the STR loci out-performed the SNP loci on a single locus basis, combinations of SNPs outperformed the STRs based upon total number of alleles. The latter observation is consistent with a recent comparison between the two marker classes in chum salmon (*Onchorhynchus keta*) [16]. Whilst the number of alleles displayed by a locus may be a good predictor of its individual assignment power [20,21,36], a trend that is also evident between marker classes when choosing loci randomly [19], the selection of highly diagnostic SNPs from larger panels has the ability to increase assignment considerably, as has been demonstrated in large human panels [8], and in the present study. Whilst it can be argued that the principle of selecting highly diagnostic loci from larger panels can also be applied to any class of marker, the continued technological advances in SNP detection genotyping platforms will favor this strategy for SNP markers.

The drop in accuracy of self-assignment conducted for the entire data set in Geneclass when adding 100-195 SNP loci based upon selection by BELS (Figure 4) represents a striking result. It is acknowledged that the peak in this curve may be "inflated" due to a combination of the fact that identification of loci was conducted without re-sampling the raw data in BELS, and, that the same individuals were used for locus identification and self-assignment. Consequently, the peak in this specific curve should be viewed with caution. Whilst it could be argued that identification of informative loci could have been performed using examination and test sub-data sets by dividing each baseline sample into two components, it is suggested that this would have nevertheless identified a very similar set of loci. Furthermore, the marker identification and self-assignment test design was chosen to examine potential gains in genetic assignment through loci selection as opposed to validate a universally applicable set of loci that would be valid for a wide range of other studies. Most importantly however, all combinations of SNP and STR markers identified sample I, which was not included in the marker identification process, as the source of the unknown escaped salmon (RF).

Although the drop in self-assignment observed over 100 SNP loci may have been linked to the conditions presented above, it is also suggested that this may be due to the inclusion of weakly and/or non-informative loci, and the manner in which Geneclass deals with such (large numbers) data. This idea is supported by the fact that no increase in self-assignment was observed from 195 to 300 loci (which did not include any locus ranking in BELS) when assigning the 40 individuals removed from the baseline with Geneclass (195 to 300 SNP loci = 78% to 80%), which contrasts with the fact that a large increase was observed using STRUCTURE (195 to 300 SNP loci = 78% to 88%). Clearly, STRUCTURE was able to utilise data from addition of extra loci whereas Geneclass was not. This may be linked with the different computation methods implemented in the programs, and therefore, this topic requires further investigation.

The ability for loci to perform individual genetic assignment can be evaluated by a number of criteria and programs, for example various distance based methods (e.g., [15], informativeness for assignment (*I*_n_) [19] and modifications of it [8]. Whilst it was not the intention of the present study to present an exhaustive comparison between various locus selection methods, the efficacy of the program BELS compared to distance based methods was clearly demonstrated. However, whilst identifying the best loci from all 300 SNPs (data not presented), BELS identified a non-optimal reduced set of loci compared to the selection carried out with 195 loci only. BELS uses a backwards locus selection algorithm (see [40]), and it is suggested that as exclusion of any single locus in the 300 loci data set would not lead to any clear change in self-assignment accuracy, the program was unable to identify the best loci when starting with such large numbers. This effect potentially existed for 195 loci also, and it cannot be excluded that a more diagnostic set of 50 or 100 loci would have been identified if one had started with the 100 loci displaying highest global F_ST _as opposed to the 195 loci. Therefore, use of the program BELS to identify a reduced panel of informative markers from a very large set of loci (several hundred or more) should be conducted with caution.

With the exception of the 28 unlinked SNP data set which overinflated the differences observed between samples (Figure 2), varying the number of SNPs and STRs included in the analyses, and the criteria upon which the loci were selected, had little influence on the overall genetic relationships among the samples, as illustrated by UPGMA diagrams (Figure 3) and Bayesian clustering of the data (additional file 3). Whilst these comparisons were by no means exhaustive, they illustrate that selection of loci displaying very high global F_ST _values represents a way to create a better discrimination between pairs of populations. For population geneticists who are interested in highly discriminatory genetic markers, the approach described here, by taking those markers displaying highest F_ST _values may provide a way to differentiate very similar populations, as is often the case for marine organisms where there is a large degree of gene flow and little differentiation.

Most statistical tests have conditions which need to be fulfilled in order to avoid violation of the underlying principles. Some of the data sets investigated in the present study, for example analysis of all 300 SNPs combined, violated some of the tests performed. The 300 SNP data set for example, included a number of markers which were tightly linked, i.e., came from the same contig. For both the individual assignments tests, and the genetic relationships among the samples however, the violations incurred in the present study did not appear to have any effect on the results of these tests, and gave more or less identical results to the data sets tested which did not violate the tests (such as the 14 STRs, 28 unlinked SNPs or 195 mapped SNPs with minimum of 1 cM distance between loci). Whilst deliberate violations of tests is not recommended, here, we computed these tests in order extract the maximum amount of information from the SNP data set as possible. However, the simulations also indicate that moderate violation of the underlying principles of genetic assignment and phylogeny may not lead to erroneous results. In a test of individual assignment, Narum et al., [15] also reported that minor violations of some of the test-conditions did not affect the results. Furthermore, in a comparison of genetic assignment using non-recombining part of the Y-chromosome, treating the data as both haplotype and multiple independent loci, which seriously violates the principles of the tests [51,52] almost no difference in assignment were observed.

Individual genetic assignment is based upon matching or excluding an individual's multilocus composite genotype to the group genetic profiles of potential source populations. A number of statistical methods to test this exist (reviewed by [53,54]). However, for some applications, such as where all potential source populations may not have been sampled, and forensics, a statistical test of the "similarity" is required. Data from this study indicate that whilst only a low number of SNP, STR or combined SNP and STR loci were required to effectively identify baseline sample I as the major source for the unknown individuals, in order to reject other baseline samples as potential sources for individual escapees, a larger number of loci were required (data not presented).

## Conclusion

Results of this study demonstrate that the identification of a highly informative set of SNPs from a larger panel gave significantly more accurate individual genetic self-assignment compared to any combination of STR loci. Furthermore, once a set of 50 or more diagnostic SNP loci were included in the self-assignment analyses, addition of even the most informative STR loci did not increase the accuracy of self-assignment, whilst addition of informative SNPs to any combination of STR loci increased self-assignment. These results clearly demonstrate that identification of highly informative SNP markers from the screening of larger pools represents a powerful approach to create molecular tools to study individual ancestry.

## Authors' contributions

KAG conceived and designed the study, supervised STR analysis, performed all statistical analysis, and wrote the first draft of the manuscript. MMH helped design the study and contributed to statistical analysis. SL contributed to the design of the study and supervised analysis of SNPs. TDA contributed to the design of the study and contributed to statistical analysis. BH contributed to the design of the study. ØS contributed to the both the conception and design of the study. All authors contributed to the writing and approved the final version of the manuscript.

## Supplementary Material

Additional file 1 **Summary statistics for 388 SNP and 15 STR loci**. Data set consists of approximately 500 farmed Atlantic salmon arranged in 10 samples/populations. Click here for file

Additional file 2 **Global F_ST _values (estimated over 9 samples/populations of Atlantic salmon) for 300 polymorphic SNP markers**. Each linkage group is represented by a single figure, with associated SNPs and their global F_ST _values plotted on them. Unmapped SNPs are ranked by global F_ST _and placed in the bottom figure. Click here for file

Additional file 3 **Assignment of individual fish to samples A-I and RF**. Figures based upon information from 14 STR loci (top), 28 unlinked SNPs (upper middle), 195 mapped SNPs (lower middle), and 300 SNPs (bottom), each for *K *= 3, 4 and 5. Click here for file

## References

[B1] Hayes B Laerdahl JK Lien S Moen T Berg P Hindar K Davidson WS Koop BF Adzhubei A Hoyheim B An extensive resource of single nucleotide polymorphism markers associated with Atlantic salmon (Salmo salar) expressed sequences Aquaculture 2007 265 1-4 82 90 10.1016/j.aquaculture.2007.01.037

[B2] Moen T Hayes B Baranski M Berg PR Kjoglum S Koop BF Davidson WS Omholt SW Lien S A linkage map of the Atlantic salmon (Salmo salar) based on EST-derived SNP markers Bmc Genomics 2008 15 9 223 10.1186/1471-2164-9-223 PMC2405805 18482444

[B3] Brumfield RT Beerli P Nickerson DA Edwards SV The utility of single nucleotide polymorphisms in inferences of population history Trends in Ecology & Evolution 2003 18 5 249 256 10.1016/S0169-5347(03)00018-1

[B4] Morin PA Luikart G Wayne RK SNPs in ecology, evolution and conservation Trends in Ecology & Evolution 2004 19 4 208 216 10.1016/j.tree.2004.01.009

[B5] Angers B Estoup A Jarne P Microsatellite size homoplasy, SSCP, and population structure: A case study in the freshwater snail Bulinus truncatus Molecular Biology and Evolution 2000 17 12 1926 1932 11110909 10.1093/oxfordjournals.molbev.a026294

[B6] Hoffman JI Amos W Microsatellite genotyping errors: detection approaches, common sources and consequences for paternal exclusion Molecular Ecology 2005 14 2 599 612 10.1111/j.1365-294X.2004.02419.x 15660949

[B7] Baric S Monschein S Hofer M Grill D Dalla Via J Comparability of genotyping data obtained by different procedures an inter-laboratory survey Journal of Horticultural Science & Biotechnology 2008 83 2 183 190

[B8] Lao O van Duijn K Kersbergen P de Knijff P Kayser M Proportioning whole-genome single-nucleotide-polymorphism diversity for the identification of geographic population structure and genetic ancestry American Journal of Human Genetics 2006 78 4 680 690 10.1086/501531 16532397 PMC1424693

[B9] Gibbs RA Taylor JF Van Tassell CP Barendse W Eversoie KA Gill CA Green RD Hamernik DL Kappes SM Lien S Genome-Wide Survey of SNP Variation Uncovers the Genetic Structure of Cattle Breeds Science 2009 324 5926 528 532 10.1126/science.1167936 19390050 PMC2735092

[B10] Van Tassell CP Smith TPL Matukumalli LK Taylor JF Schnabel RD Lawley CT Haudenschild CD Moore SS Warren WC Sonstegard TS SNP discovery and allele frequency estimation by deep sequencing of reduced representation libraries Nat Methods 2008 5 3 247 252 10.1038/nmeth.1185 18297082

[B11] McKay SD Schnabel RD Murdoch BM Matukumalli LK Aerts J Coppieters W Crews D Dias E Gill CA Gao C An assessment of population structure in eight breeds of cattle using a whole genome SNP panel Bmc Genetics 2008 9 9 10.1186/1471-2156-9-37 18492244 PMC2408608

[B12] Seddon JM Parker HG Ostrander EA Ellegren H SNPs in ecological and conservation studies: a test in the Scandinavian wolf population Molecular Ecology 2005 14 2 503 511 10.1111/j.1365-294X.2005.02435.x 15660941

[B13] Rengmark AH Slettan A Skaala O Lie O Lingaas F Genetic variability in wild and farmed Atlantic salmon (Salmo salar) strains estimated by SNP and microsatellites Aquaculture 2006 253 1-4 229 237 10.1016/j.aquaculture.2005.09.022

[B14] Smith CT Antonovich A Templin WD Elfstrom CM Narum SR Seeb LW Impacts of marker class bias relative to locus-specific variability on population inferences in Chinook salmon: A comparison of single-nucleotide polymorphisms with short tandem repeats and allozymes Transactions of the American Fisheries Society 2007 136 6 1674 1687 10.1577/T06-227.1

[B15] Narum SR Banks M Beacham TD Bellinger MR Campbell MR Dekoning J Elz A Guthrie CM Kozfkay C Miller KM Differentiating salmon populations at broad and fine geographical scales with microsatellites and single nucleotide polymorphisms Molecular Ecology 2008 17 15 3464 3477 19160476 10.1111/j.1365-294x.2008.03851.x

[B16] Smith CT Seeb LW Number of alleles as a predictor of the relative assignment accuracy of short tandem repeat (STR) and single-nucleotide-polymorphism (SNP) baselines for chum salmon Transactions of the American Fisheries Society 2008 137 3 751 762 10.1577/T07-104.1

[B17] Ryynanen HJ Tonteri A Vasemagi A Primmer CR A comparison of biallelic markers and microsatellites for the estimation of population and conservation genetic parameters in Atlantic salmon (Salmo salar) Journal of Heredity 2007 98 7 692 704 10.1093/jhered/esm093 17986472

[B18] Morin PA Martien KK Taylor BL Assessing statistical power of SNPs for population structure and conservation studies Molecular Ecology Resources 2009 9 1 66 73 10.1111/j.1755-0998.2008.02392.x 21564568

[B19] Rosenberg NA Li LM Ward R Pritchard JK Informativeness of genetic markers for inference of ancestry American Journal of Human Genetics 2003 73 6 1402 1422 10.1086/380416 14631557 PMC1180403

[B20] Kalinowski ST Genetic polymorphism and mixed-stock fisheries analysis Canadian Journal of Fisheries and Aquatic Sciences 2004 61 7 1075 1082 10.1139/f04-060

[B21] Beacham TD Candy JR McIntosh B MacConnachie C Tabata A Kaukinen K Deng LT Miller KM Withler RE Varnavskaya N Estimation of stock composition and individual identification of sockeye salmon on a Pacific Rim basis using microsatellite and major histocompatibility complex variation Transactions of the American Fisheries Society 2005 134 5 1124 1146 10.1577/T05-005.1

[B22] Glover KA Genetic characterisation of farmed rainbow trout in Norway: intra- and inter-strain variation reveals potential for identification of escapees Bmc Genetics 2008 16 9 87 10.1186/1471-2156-9-87 PMC2640418 19087266

[B23] Spencer PBS Hampton JO Illegal translocation and genetic structure of feral pigs in Western Australia Journal of Wildlife Management 2005 69 1 377 384 10.2193/0022-541X(2005)069<0377:ITAGSO>2.0.CO;2

[B24] Frantz AC Pourtois JT Heuertz M Schley L Flamand MC Krier A Bertouille S Chaumont F Burke T Genetic structure and assignment tests demonstrate illegal translocation of red deer (Cervus elaphus) into a continuous population Molecular Ecology 2006 15 11 3191 3203 10.1111/j.1365-294X.2006.03022.x 16968264

[B25] Kotze A Ehlers K Cilliers DC Grobler JP The power of resolution of microsatellite markers and assignment tests to determine the geographic origin of cheetah (Acinonyx jubatus) in Southern Africa Mammalian Biology 2008 73 6 457 462 10.1016/j.mambio.2007.10.011

[B26] Primmer CR Koskinen MT Piironen J The one that did not get away: individual assignment using microsatellite data detects a case of fishing competition fraud Proceedings of the Royal Society B-Biological Sciences 2000 267 1453 1699 1704 10.1098/rspb.2000.1197 PMC1690726 11467434

[B27] Glover KA Skilbrei OT Skaala O Genetic assignment identifies farm of origin for Atlantic salmon Salmo salar escapees in a Norwegian fjord Ices Journal of Marine Science 2008 65 6 912 920 10.1093/icesjms/fsn056

[B28] Schrey AW Sloss BL Sheehan RJ Heidinger RC Heist EJ Genetic discrimination of middle Mississippi River Scaphirhynchus sturgeon into pallid, shovelnose, and putative hybrids with multiple microsatellite loci Conservation Genetics 2007 8 3 683 693 10.1007/s10592-006-9215-9

[B29] McCusker MR Paterson IG Bentzen P Microsatellite markers discriminate three species of North Atlantic wolffishes (Anarhichas spp.) Journal of Fish Biology 2008 72 2 375 385 10.1111/j.1095-8649.2007.01701.x

[B30] Baarøy V Gjerde B Heggberget TG Jensen PE Maroni K Sandvik S Skaala Ø Identifisering av rømt oppdrettlaks. Utredning av utvalg nedsatt av Fiskeridirektøren (Identification of escaped farmed salmon. Report from the committee to the Directer of Fisheries) Norwegian 2008 55

[B31] Paterson S Piertney SB Knox D Gilbey J Verspoor E Characterization and PCR multiplexing of novel highly variable tetranucleotide Atlantic salmon (Salmo salar L.) microsatellites Molecular Ecology Notes 2004 4 2 160 162 10.1111/j.1471-8286.2004.00598.x

[B32] O'Reilly PT Hamilton LC McConnell SK Wright JM Rapid analysis of genetic variation in Atlantic salmon (Salmo salar) by PCR multiplexing of dinucleotide and tetranucleotide microsatellites Canadian Journal of Fisheries and Aquatic Sciences 1996 53 10 2292 2298 10.1139/cjfas-53-10-2292

[B33] King TL Eackles MS Letcher BH Microsatellite DNA markers for the study of Atlantic salmon (Salmo salar) kinship, population structure, and mixed-fishery analyses Molecular Ecology Notes 2005 5 1 130 132 10.1111/j.1471-8286.2005.00860.x

[B34] McConnell SK Oreilly P Hamilton L Wright JN Bentzen P Polymorphic Microsatellite loci from Atlantic salmon (Salmo-salar) - genetic differentiation of North-American and European Populations Canadian Journal of Fisheries and Aquatic Sciences 1995 52 9 1863 1872 10.1139/f95-779

[B35] Sanchez JA Clabby C Ramos D Blanco G Flavin F Vazquez E Powell R Protein and microsatellite single locus variability in Salmo salar L (Atlantic salmon) Heredity 1996 77 423 432 10.1038/hdy.1996.162 8885382

[B36] Glover KA Hansen MM Skaala O Identifying the source of farmed escaped Atlantic salmon (Salmo salar): Bayesian clustering analysis increases accuracy of assignment Aquaculture 2009 290 1-2 37 46 10.1016/j.aquaculture.2009.01.034

[B37] Pompanon F Bonin A Bellemain E Taberlet P Genotyping errors: Causes, consequences and solutions Nature Reviews Genetics 2005 6 11 847 859 10.1038/nrg1707 16304600

[B38] Lorenz S Brenna-Hansen S Moen T Roseth A Davidson WS Omholdt S Lien S BAC-based upgrading and physical integration of a genetic SNP map in Atlantic salmon Animal Genetics 2009 in press 19917045 10.1111/j.1365-2052.2009.01963.x

[B39] Tang K Fu DJ Julien D Braun A Cantor CR Koster H Chip-based genotyping by mass spectrometry Proceedings of the National Academy of Sciences of the United States of America 1999 96 18 10016 10020 10.1073/pnas.96.18.10016 10468554 PMC17834

[B40] Bromaghin JF BELS: backward elimination locus selection for studies of mixture composition or individual assignment Molecular Ecology Resources 2008 8 3 568 571 10.1111/j.1471-8286.2007.02010.x 21585834

[B41] Dieringer D Schlotterer C MICROSATELLITE ANALYSER (MSA): a platform independent analysis tool for large microsatellite data sets Molecular Ecology Notes 2003 3 1 167 169 10.1046/j.1471-8286.2003.00351.x

[B42] Excoffier L Laval G Schneider S Arlequin (version 3.0): An integrated software package for population genetics data analysis Evolutionary Bioinformatics 2005 1 47 50 PMC2658868 19325852

[B43] Tamura K Dudley J Nei M Kumar S MEGA4: Molecular evolutionary genetics analysis (MEGA) software version 4.0 Molecular Biology and Evolution 2007 24 8 1596 1599 10.1093/molbev/msm092 17488738

[B44] Takezaki N Rzhetsky A Nei M Phylogenetic test of the molecular clock and linearized trees Molecular Biology and Evolution 1995 12 5 823 833 7476128 10.1093/oxfordjournals.molbev.a040259

[B45] Cornuet JM Piry S Luikart G Estoup A Solignac M New methods employing multilocus genotypes to select or exclude populations as origins of individuals Genetics 1999 153 4 1989 2000 10581301 10.1093/genetics/153.4.1989 PMC1460843

[B46] Rannala B Mountain JL Detecting immigration by using multilocus genotypes Proceedings of the National Academy of Sciences of the United States of America 1997 94 17 9197 9201 10.1073/pnas.94.17.9197 9256459 PMC23111

[B47] Pritchard JK Stephens M Donnelly P Inference of population structure using multilocus genotype data Genetics 2000 155 2 945 959 10835412 10.1093/genetics/155.2.945 PMC1461096

[B48] Falush D Stephens M Pritchard JK Inference of population structure using multilocus genotype data: Linked loci and correlated allele frequencies Genetics 2003 164 4 1567 1587 12930761 10.1093/genetics/164.4.1567 PMC1462648

[B49] Foll M Gaggiotti O A Genome-Scan Method to Identify Selected Loci Appropriate for Both Dominant and Codominant Markers: A Bayesian Perspective Genetics 2008 180 2 977 993 10.1534/genetics.108.092221 18780740 PMC2567396

[B50] Nei M Estimation of average heterozygosity and genetic distance from a small number of individuals Genetics 1978 89 3 583 590 17248844 10.1093/genetics/89.3.583 PMC1213855

[B51] Als TD Jorgensen TH Borglum AD Petersen PA Mors O Wang AG Highly discrepant proportions of female and male Scandinavian and British Isles ancestry within the isolated population of the Faroe Islands European Journal of Human Genetics 2006 14 4 497 504 10.1038/sj.ejhg.5201578 16434998

[B52] Jorgensen TH Buttenschon HN Wang AG Als TD Borglum AD Ewald H The origin of the isolated population of the Faroe Islands investigated using Y chromosomal markers Human Genetics 2004 115 1 19 28 10.1007/s00439-004-1117-7 15083358

[B53] Hansen MM Kenchington E Nilsen EE Assigning individual fish to population using microsatellite DNA markers Fish and Fisheries 2001 2 93 112 10.1046/j.1467-2960.2001.00043.x

[B54] Manel S Gaggiotti OE Waples RS Assignment methods: matching biological questions with appropriate techniques Trends in Ecology & Evolution 2005 20 3 136 142 10.1016/j.tree.2004.12.004 16701357

